# Single-Operator Peroral Cholangioscopy for Extraction of Cystic Duct Stones in Postcholecystectomy Mirizzi Syndrome

**DOI:** 10.1155/2017/1710501

**Published:** 2017-01-22

**Authors:** Jason Deforest Jones, Rishi Pawa

**Affiliations:** Division of Gastroenterology, Department of Internal Medicine, Wake Forest School of Medicine, Winston-Salem, NC, USA

## Abstract

Mirizzi syndrome is an exceptionally rare diagnosis with an annual incidence of less than 1% in developed countries. In this disease process, stone burden in the cystic duct or gallbladder neck leads to common hepatic duct obstruction, either by mechanical compression or secondary inflammation. Mirizzi syndrome is classified into one of four types based on the presence and severity of cholecystobiliary fistulization. Treatment is primarily surgical in nature and largely dictated by the type of Mirizzi syndrome encountered. It is typically diagnosed in the preoperative or operative setting of cholecystectomy; however, there have been rare occurrences of postcholecystectomy diagnosis. Factors thought to predispose to postcholecystectomy disease include low insertion of the cystic duct and long remnant duct length. Few case reports exist describing this phenomenon and its management, which is made exceptionally difficult due to the presence of inflammation and surgical adhesion. We present the case of a young female with postcholecystectomy Mirizzi syndrome who underwent successful endoscopic management using peroral cholangioscopy and electrohydraulic lithotripsy. We also provide a brief overview of both Mirizzi syndrome and peroral cholangioscopy.

## 1. Introduction

Mirizzi syndrome is defined as common hepatic duct obstruction caused by extrinsic compression or inflammation from cystic duct or gallbladder neck stone burden. The syndrome is quite rare, especially in patients with prior cholecystectomy, in whom only a small amount of case reports have been published. The management of cystic duct stones in these patients with prior cholecystectomy can prove difficult and often requires surgical intervention. Peroral cholangioscopy using direct visualization and electrohydraulic lithotripsy (EHL) offers an alternative management solution which gives a single-operator the ability to detect strictures and remove large stones in the pancreaticobiliary system which are otherwise not amenable to endoscopic retrograde cholangiopancreatography (ERCP). We present a case of the successful use of cholangioscopy and EHL for endoscopic visualization and management of a cystic duct stone causing Mirizzi syndrome in a postcholecystectomy patient.

## 2. Case Presentation

**Figure 1:**
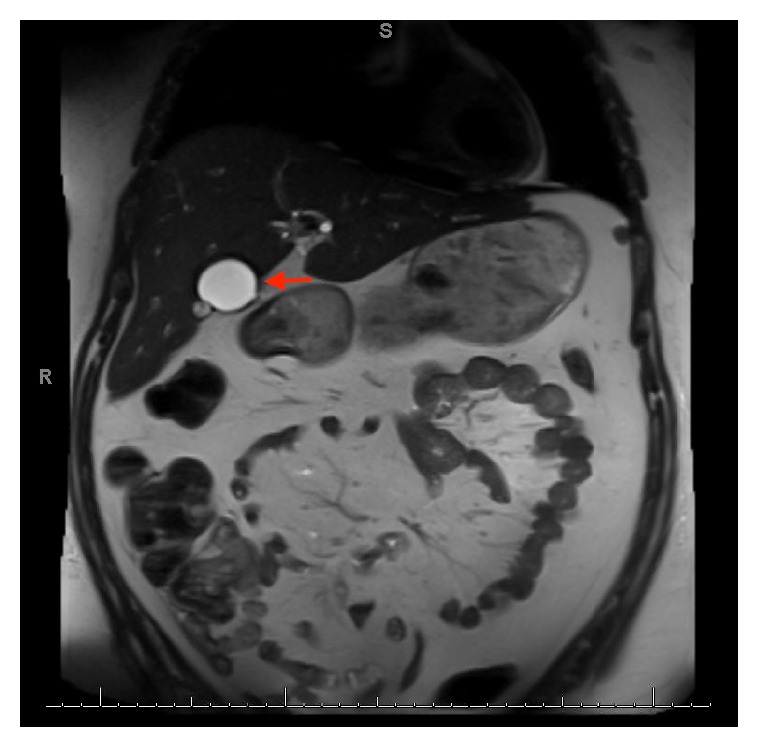
Preintervention MRCP revealing a fluid collection in continuity with the cystic duct representative of a contained bile leak (marked with arrow).

**Figure 2:**
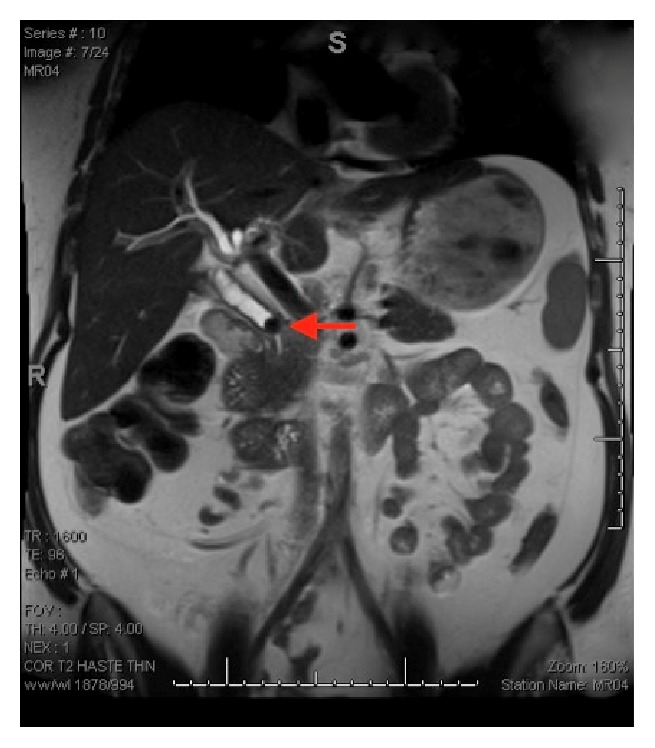
Preintervention MRCP revealing a nine-millimeter stone in the cystic duct (marked with arrow).

**Figure 3:**
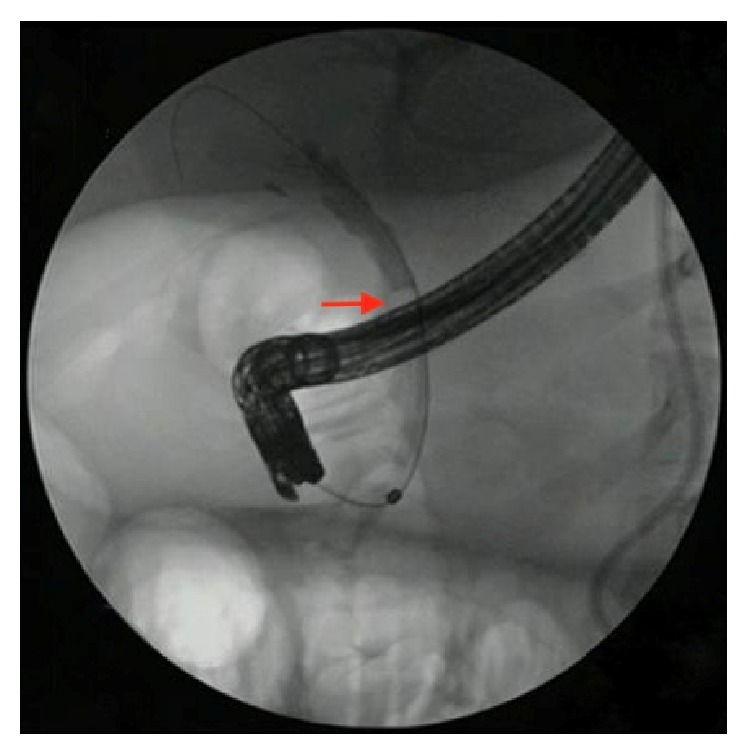
ERCP revealing a stone in the cystic duct (marked with arrow).

**Figure 4:**
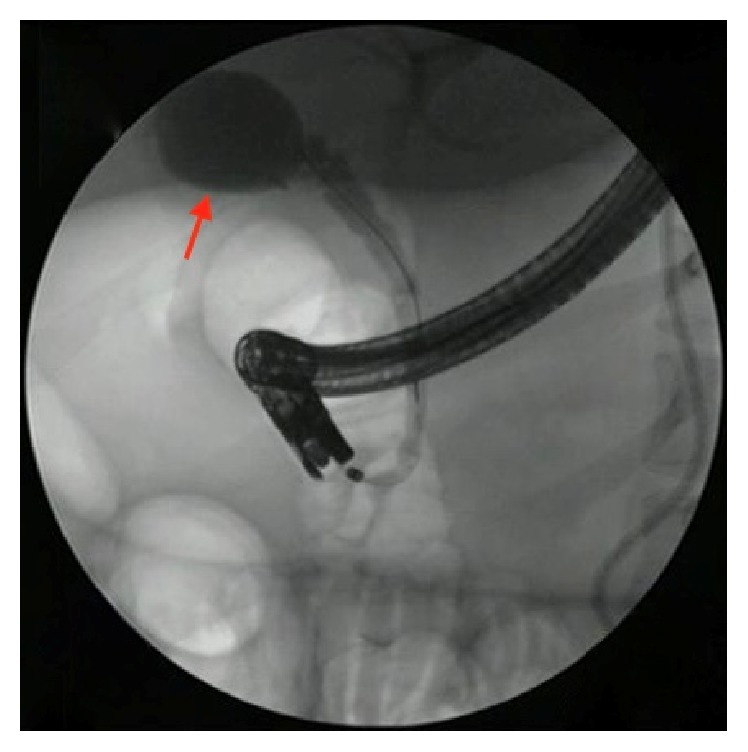
ERCP revealing proximal cystic duct dilatation and extravasation of contrast suggestive of a contained bile leak in this patient with a history of cholecystectomy. The contained bile leak is marked with an arrow.

**Figure 5:**
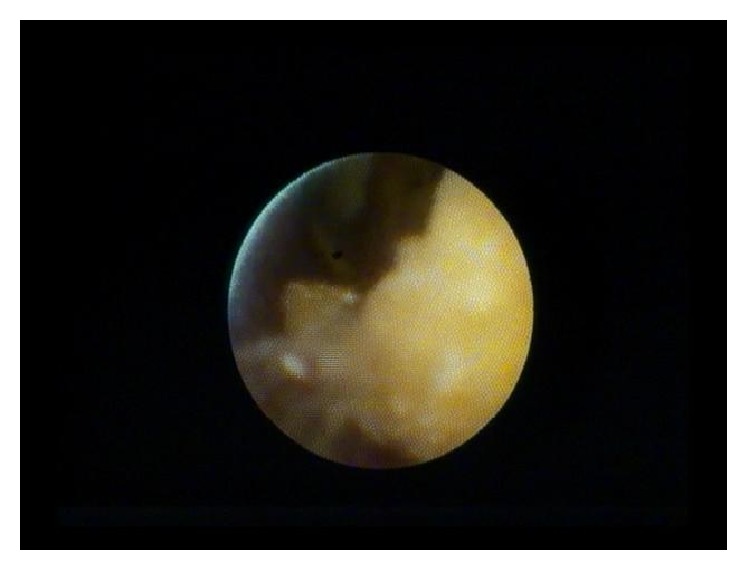
Nine-millimeter cystic duct stone visualized using peroral cholangioscopy.

**Figure 6:**
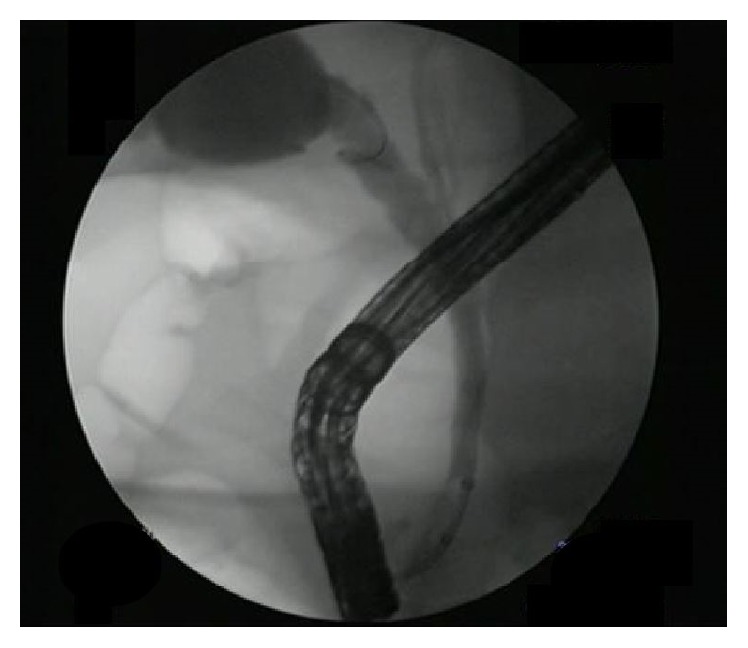
Occlusion cholangiogram after cystic duct stone removal.

**Figure 7:**
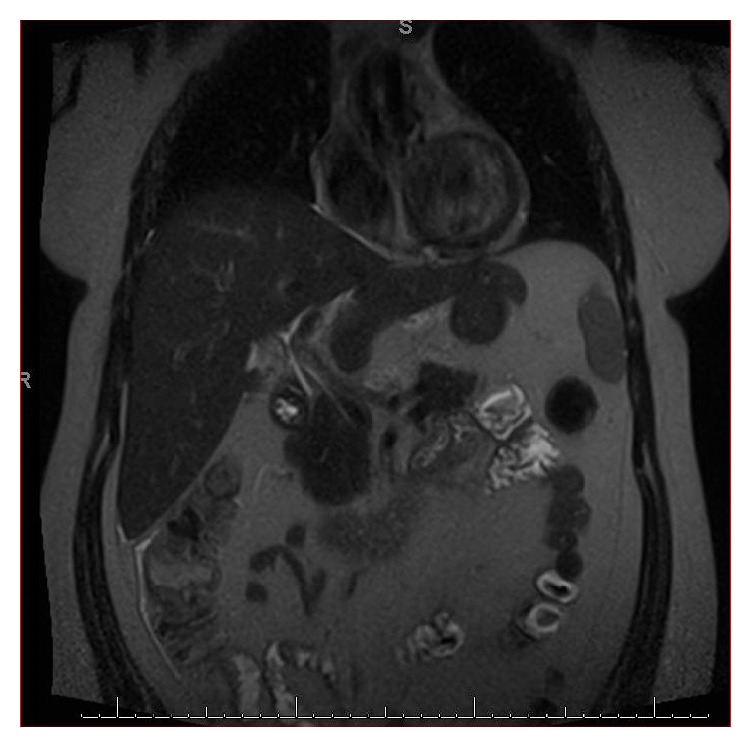
Postintervention MRCP revealing stone extraction and resolution of previously seen fluid collection.

A 27-year-old female with a history of cholecystectomy for symptomatic cholelithiasis three years prior to admission presented with epigastric pain radiating to the right upper quadrant and back. Initial laboratory tests on presentation were a total bilirubin (TBili) 5.5 mg/dL, alkaline phosphatase (ALP) 288 U/L, aspartate aminotransferase (AST) 316 U/L, and alanine aminotransferase (ALT) 394 U/L, and magnetic resonance cholangiopancreatography (MRCP) revealed a fluid collection in continuity with the cystic duct and a nine-millimeter stone at the confluence of the cystic and common hepatic duct, resulting in both biliary and cystic duct dilatation with adjacent inflammation (Figures 1 and 2). Using ERCP, the cystic duct was cannulated and a filling defect consistent with a nine-millimeter stone was confirmed, along with proximal cystic duct dilatation and extravasation of contrast suggestive of a contained bile leak (Figures 3 and 4). Following failed attempts at balloon-assisted stone extraction, cholangioscopy was performed. A single therapeutic endoscopist who has performed >30 interventions using the SpyGlass system was the primary operator and the case was performed under general anesthesia. A Pentax ED-3490TK therapeutic duodenoscope (Pentax Medical; Tokyo, Japan) was used to facilitate passage of the SpyGlass system. The 10 Fr SpyGlass cholangioscope measuring 250 cm in length was then used to directly visualize the stone in the cystic duct (Figure 5). The visualized stone was irrigated using saline prior to EHL, which was achieved using a Nortech Autolith system (Northgate Technologies Inc.; Elgin, IL) and a 1.9 Fr EHL probe. Power was set to 90 W with a rate of 3 pulsations/second, the latter of which was escalated as needed to achieve stone fragmentation. Subsequent balloon sweeps of the cystic duct successfully cleared stone fragments (Figure 6) and repeat MRCP (Figure 7) one year after hospital discharge revealed no evidence of fluid collection or stone in the cystic duct.

## 3. Discussion

Mirizzi syndrome is a rare entity generally encountered in either the preoperative or intraoperative stages of cholecystectomy, with few case reports citing postcholecystectomy disease. Increased remnant cystic duct length and low insertion of the duct are hypothesized to predispose to postcholecystectomy Mirizzi syndrome. It is classified into one of four categories based on the development and severity of cholecystobiliary fistulization. Type I Mirizzi syndrome, as described in the aforementioned case, involves extrinsic compression of the common hepatic duct secondary to gallbladder neck or cystic duct stone impaction [1]. Disease pathology can also intermittently occur as a sequela of inflammation. Cholecystobiliary fistula involvement of one-third of the CBD circumference, two-thirds of the CBD circumference, and fistula development leading to complete common hepatic duct wall destruction is classified as types II, III, and IV, respectively [2].

The typical clinical presentation of Mirizzi syndrome is obstructive jaundice and a cholestatic injury pattern on laboratory analysis, sometimes with evidence of cholangitis [3]. MRCP is the imaging modality of choice to obtain a diagnosis [4]. As previously mentioned, Mirizzi syndrome is most commonly discovered in the preoperative evaluation of patients with cholelithiasis preparing to undergo cholecystectomy. Antoniou et al. found a decrease in surgical conversion, procedural complications, and need for reoperation in patients diagnosed with Mirizzi syndrome in the preoperative setting, thus emphasizing the importance of consideration of this condition [5]. Surgery is the most common treatment modality for Mirizzi syndrome, with the specific approach based on the type of disease present, technical skill of the surgeon, and the perioperative setting in which the disease is discovered. The cystic duct stones found in Mirizzi syndrome lead to a large inflammatory response which makes surgical dissection of the triangle of Calot quite difficult; thus one can expect this to be further complicated in patients who are status postcholecystectomy, in which the surgeon is faced with both surgical adhesion and severe inflammation [1, 6]. Type I Mirizzi syndrome is typically managed via open or laparoscopic cholecystectomy, either total or subtotal [1]. As expected, the presence of a cholecystobiliary fistula in types II–IV necessitates a more complicated approach to treatment. Type II Mirizzi syndrome requires subtotal cholecystectomy with bile duct reconstruction using residual gallbladder wall [1, 7]. Lastly, treatment for Mirizzi syndrome types III and IV relies on bilioenteric anastomosis [1].

Due to the need for management solutions in poor surgical candidates, as well as the inherent risk of bile duct injury associated with surgery in Mirizzi syndrome, several endoscopic and percutaneous approaches to treatment have been explored. Endoscopic therapy has been reported with the use of direct stone removal via balloon or basket as well as electrohydraulic and laser lithotripsy [3, 8, 9]. Cystic duct stump cannulation is accomplished using either fluoroscopy or direct cholangioscopy visualization. We have traditionally attempted initial cannulation using fluoroscopic guidance; however, when this is not possible a cholangioscope is introduced to localize the cystic duct stump opening and subsequently cannulate using direct visualization. Bhandari et al. recently reported a 94% success rate in single session cholangioscopy-guided laser lithotripsy in the setting of endoscopic or surgical failure [10]. Peroral cholangioscopy, while once plagued by several limitations such as restricted maneuverability, cumbersome setup, expensive maintenance, and a dual operator requirement, has become a nonsurgical, safe, and effective treatment modality with various indications in biliary pathology [11]. Boston Scientific Corporation's SpyGlass direct visualization system (Boston Scientific, Marlborough, MA) was used in this case report. SpyGlass-directed therapy has been shown to have success rates of 90–100% in the treatment of biliary stones [11]. Stone removal using the SpyGlass system is accomplished using either EHL or laser lithotripsy, with EHL shown to be superior to the more traditionally utilized extracorporeal shock wave lithotripsy [12, 13]. Complication rates of peroral cholangioscopy using the SpyGlass system are low. The most common complication reported has been acute cholangitis, with an incidence of approximately 3%, while the incidence rate of other complications such as abdominal pain, bacteremia, and pancreatitis tends to mimic those one can experience with routine ERCP [11].

Postcholecystectomy Mirizzi syndrome is an exceptionally rare phenomenon with a limited number of case reports currently available to describe the process and its complicated management. Although rare, one would expect the incidence of postcholecystectomy Mirizzi syndrome to increase as a direct result of the predominance of laparoscopic cholecystectomies in surgical practice, in which the target of surgical dissection is the junction between the cystic duct and gallbladder, thus predisposing to an increase in cystic duct stump length. Conventional balloon passage failed in our case due to large stone size in relation to duct size. No adverse events were experienced in the presented case. We therefore conclude that endoscopic management of postcholecystectomy Mirizzi syndrome can provide the patient with a safe, minimally invasive treatment modality for a problem which typically requires surgical intervention.
